# *Medicago truncatula *contains a second gene encoding a plastid located glutamine synthetase exclusively expressed in developing seeds

**DOI:** 10.1186/1471-2229-10-183

**Published:** 2010-08-19

**Authors:** Ana R Seabra, Cristina P Vieira, Julie V Cullimore, Helena G Carvalho

**Affiliations:** 1 Instituto de Biologia Molecular e Celular da Universidade do Porto, Rua do Campo Alegre, 823, 4150-180 Porto, Portugal; 2 Laboratoire des Interactions Plantes-Microorganismes, Institut National de la Recherche Agronomique - Centre National de la Recherche Scientifique, Boite Postale 52627, 31326 Castanet-Tolosan Cedex, France

## Abstract

**Background:**

Nitrogen is a crucial nutrient that is both essential and rate limiting for plant growth and seed production. Glutamine synthetase (GS), occupies a central position in nitrogen assimilation and recycling, justifying the extensive number of studies that have been dedicated to this enzyme from several plant sources. All plants species studied to date have been reported as containing a single, nuclear gene encoding a plastid located GS isoenzyme per haploid genome. This study reports the existence of a second nuclear gene encoding a plastid located GS in *Medicago truncatula*.

**Results:**

This study characterizes a new, second gene encoding a plastid located glutamine synthetase (GS2) in *M. truncatula*. The gene encodes a functional GS isoenzyme with unique kinetic properties, which is exclusively expressed in developing seeds. Based on molecular data and the assumption of a molecular clock, it is estimated that the gene arose from a duplication event that occurred about 10 My ago, after legume speciation and that duplicated sequences are also present in closely related species of the Vicioide subclade. Expression analysis by RT-PCR and western blot indicate that the gene is exclusively expressed in developing seeds and its expression is related to seed filling, suggesting a specific function of the enzyme associated to legume seed metabolism. Interestingly, the gene was found to be subjected to alternative splicing over the first intron, leading to the formation of two transcripts with similar open reading frames but varying 5' UTR lengths, due to retention of the first intron. To our knowledge, this is the first report of alternative splicing on a plant GS gene.

**Conclusions:**

This study shows that *Medicago truncatula *contains an additional GS gene encoding a plastid located isoenzyme, which is functional and exclusively expressed during seed development. Legumes produce protein-rich seeds requiring high amounts of nitrogen, we postulate that this gene duplication represents a functional innovation of plastid located GS related to storage protein accumulation exclusive to legume seed metabolism.

## Background

Nitrogen is a crucial nutrient that is both essential and rate limiting for plant growth and seed production. Nitrogen is assimilated in plants through the action of Glutamine Synthetase (GS, EC 6.3.1.2) forming glutamine, which serves as a building block for all nitrogen containing compounds in the plant. GS is a complex and highly regulated enzyme, which in addition to the primary ammonium assimilation, is involved in the reassimilation of ammonium released by a number of biochemical processes such as photorespiration, protein catabolism, deamination of amino acids and some specific biosynthetic reactions such as those involving methionine, isoleucine, phenylpropanoid and lignin [1]. Being the first enzyme in the main pathway of ammonium assimilation in higher plants, GS potentially represents a key component of plant nitrogen use efficiency (NUE) and yield and therefore, an extensive number of studies have been dedicated to understand how GS is regulated and how it is involved in the regulation of nitrogen metabolism in plants [1].

Legumes can obtain a significant part of their nitrogen from the atmosphere through a symbiotic interaction with nitrogen fixing bacteria. Perhaps due to this special source of nitrogen, legumes produce protein-rich seeds with a high nutritive value, representing a major source of nutrients for humans and animal livestock. Because of the ecological, nutritional and economic importance of legume seeds, the biochemical and molecular processes underlying their development have been the focus of much research in recent years. With the development of genomic resources for *Medicago truncatula*, recently this model legume has been chosen for an integrative approach toward understanding seed physiology and great advances have been made in understanding the metabolic control of seed filling and the regulatory network underlying reserve accumulation [2-5]. An original finding raised by these studies is that the genes involved in amino acid metabolism are among the most highly regulated in the seeds of *M. truncatula *[3]. The accumulation of storage proteins in seeds, involves N-remobilization from vegetative organs, a process in which GS is likely a key regulator, but although the enzyme has been thoroughly investigated in several organs of different plant species, and especially in legumes, it has been poorly investigated in seeds.

A number of GS isoenzymes has been identified in plants and classified according to the sub cellular localization as cytosolic (GS1) and plastidic (GS2), which are assumed to play non-overlapping roles. GS1 isoenzymes are involved in nitrogen assimilation and recycling derived from several different physiological processes (reviewed in [6]), whereas GS2 has been mainly implicated in the reassimilation of the ammonia released during photorespiration [7,8]. Genetic studies revealed that GS2 is encoded by a single gene per haploid genome, whereas several genes encode cytosolic polypeptides [9-16]. Functional GS2 allelic genes have been reported in the amphidiploid tobacco [17], the tetraploid alfalfa [18] and the hexaploid wheat [19]. There is no evidence for the existence of multiple GS2 genes in any plant species and clearly, *Arabidopsis thaliana *and rice, two plants whose genomes have been fully sequenced, contain a single gene encoding GS2.

*M. truncatula *provides an excellent model system for the study of GS, as it contains a very small GS gene family. Only three expressed GS genes have been reported: *MtGS1a *and *MtGS1b *encoding cytosolic polypeptides and *MtGS2 *encoding a plastid located enzyme [12,20,21]. However the genome of *M. truncatula *has been reported to contain two additional genes related to GS, the pseudogene *MtGS1c *which was found not to be expressed [21] and a prokaryotic type GSI-like gene, whose function is unknown [22]. This study reports the existence of a new gene encoding a second plastid located isoenzyme in *Medicago truncatula*. The gene was identified following the development of the genomic resources for *M. truncatula *[23] and is characterized in this article. Evidence is presented that the gene arose from a duplication event which occurred after legume speciation and that it produces a functional enzyme, exclusively expressed in developing seeds and particularly during seed filling.

## Results

### Identification and characterization of a new gene encoding a plastid-located glutamine synthetase in *Medicago truncatula*

**Figure 1:**
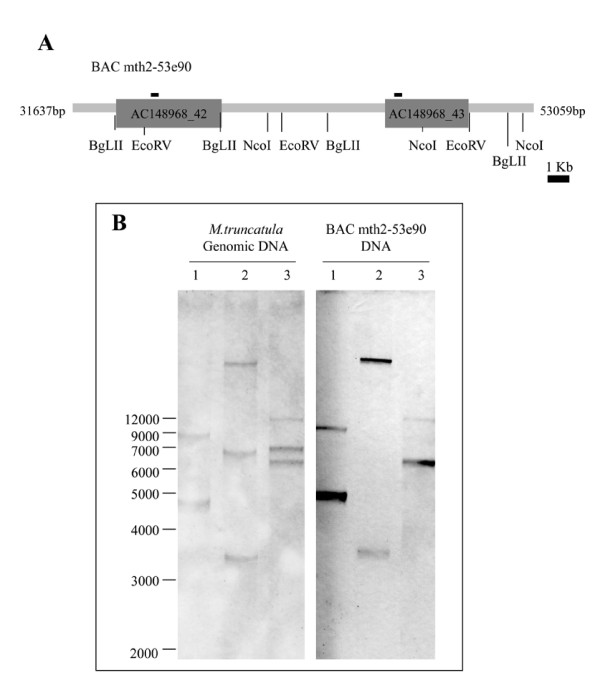
**Southern blot analysis of GS2 genes in *Medicago truncatula***. A. Schematic representation of BAC mth2-53e90 indicating the position of *MtGS2a *(AC148968-43) and *MtGS2b *(AC 1448968-42) and the restriction sites relevant for the southern analysis. The position of the probe used for the Southern analysis is also indicated. B. Southern hybridization of *M. truncatula *J5 genomic DNA and BAC mth2-53e90 DNA. 20 μg of genomic DNA and 5 μg of BAC DNA were digested with *BgLII *(1), *NcoI *(2) and *EcoRV *(3) and probed with a 260 bp DNA fragment corresponding to part of the 5'UTR and coding sequence of GS2a cDNA.

Following the advances in *M. truncatula *genome sequencing, a new gene encoding a plastid located GS was identified using the available genomic sequence data from the large-scale genome assembly for this model plant [23]. This newly identified gene is located on chromosome 2 approximately 8 kbp downstream of the previously characterized *MtGS2 *gene (GenBank accession number: AY225150.1) and both genes are comprised within *M. truncatula *BAC mth2-53e20 (AC148968) (Figure 1A). The existence of a second plastid-located GS (GS2) gene in the plant genome was confirmed by southern blot analysis of *M. truncatula *genomic DNA, under stringent hybridization conditions, using a 260 bp *MtGS2a *fragment corresponding to the beginning of the coding region, as a probe (Figure 1B). Restriction digestions of BAC mth2-53e20, containing the two *MtGS2 *genes were run in parallel for comparison. The expected hybridization fragments (6227 bp and 8906 bp for *EcoR*V digestion, 4846 bp and 7785 bp for *Bgl*II, 3536 bp and > 1.2 kbp for *Nco*I) were detected in both the BAC and the genomic DNA (Figure 1B). Two additional hybridizing fragments, corresponding to partial digestion of the gDNA, of approximately 7000 bp and 6500 bp were detected after digestion with *Eco*RV and *Nco*I, respectively (Figure 1B).

**Figure 2:**
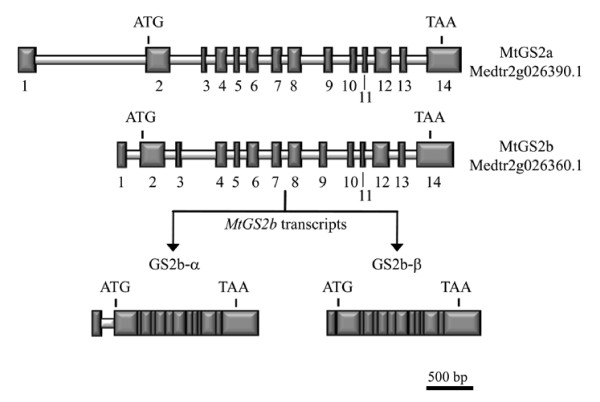
**Exon/intron organization of the two *Medicago truncatula *GS2 genes**. Exons are numbered and represented by dark gray vertical boxes. The two *MtGS2b *alternatively spliced transcripts are shown, highlighting the retention of the first intron in the *MtGS2b-α *transcript. The start (ATG) and the stop codon (TAA) are indicated.

Comparison of the genomic sequences of the two genes revealed a high degree of homology both at the level of intron/exon organization and sequence conservation (Figure 2). The two genes are composed of 14 exons (with varying lengths from 40 to 382 bp), interrupted by 13 introns (from 81 to 1201 bp). The start codon is located in the second exon and the stop codon in the last exon (Figure 2). The two genes are highly homologous. For the coding region the level of synonymous divergence per synonymous site (*K_s_*) is 0.1541 and the level of non-synonymous divergence per non-synonymous site (*K_a_*) is 0.0274. For the introns *K_s _*is 0.2646. The newly found gene contains the information to encode a functional GS2 protein with a calculated molecular weight (MW) of 47212 Da, and theoretical isoelectric point (pI) of 6.68 (ProtParam-Expasy tools [24]). The deduced protein contains a target peptide and is predicted to be driven to the plastid (score 0.911, TargetP [25]). A comparison of the deduced amino acid sequences of the two *M. truncatula *GS2 polypeptides, reveals a high degree of homology (94% amino acid identity). The existence of a second GS2 gene in the genome of *Medicago truncatula *compels a redenomination of the previously identified *MtGS2 *gene (GenBank accession number: AY225150.1), which will be referred from now on as *MtGS2a*, and designate the second gene as *MtGS2b*. The genes *MtGS2a *and *MtGS2b *are annotated in the genome as Medtr2g026390.1 and Medtr2g026360.1, respectively.

### Estimation of the time of the *M. truncatula *GS2 duplication event

The existence of a second GS2 gene in the genome of *M. truncatula*, located in the same chromosome and not too distant from the first gene suggests a recent duplication event. Therefore the age of separation of different species of the vicioide subclade was estimated and these estimates used for dating the duplication event, assuming a molecular clock. Furthermore, a search for the existence of a second GS2 gene in species where the duplication is expected to exist was performed.

Estimates for the age of separation of different species can be obtained, assuming a molecular clock [26], using the levels of synonymous divergence per synonymous site (*K_s_*) for published chloroplast genes, and as calibration point the 34 My estimate for the split between *Pisum *and *Albizia *species [27]. In Table 1, the estimated ages (in My) for the separation of four species (representing different vicioide lineages; described in figure five in [28]) from *M. truncatula *is presented. *M. truncatula *and *P. sativum *are species diverging about 14.9 My. Using the silent site divergence at the coding region of *MtGS2a *between these two species (*K_s _*= 0.2475), and the estimated age of 14.9 My as calibration point, it is inferred that the *Medicago GS2 *gene duplication (*K_s _*= 0.1668) occurred about 10 My ago. Therefore, taking into consideration the estimated ages for the separation of the different species from *M. truncatula *(Table 1), the two genes are expected to be present in *Melilotus *but not in more distantly related species. It is thus not surprising that a Blastp of the *L. japonicus *(a species of the Robinioids clade) genome at [29], using *M. truncatula MtGS2a *sequence (AY225150) as a query, allows the detection of three GS genes (chr6.CM0014.300, chr2.LjT01H08.60, and LjT32D02.120). When these sequences together with *M. truncatula *GS1 and GS2 gene sequences are used to construct a phylogeny, the two first entries cluster with *M. truncatula *GS1 genes and the latter sequence clusters with *M. truncatula *GS2 gene (data not shown). Using the same methodology, *L. japonicus *is estimated to have diverged from *M. truncatula *about 29 My ago (data not shown). It should be noted that, if a different calibration point is used, different ages will be obtained for the gene duplication and species divergence. Nevertheless, our conclusion that this gene duplication is of a recent origin and should only be present in *M. truncatula *closely related species is valid.

**Table 1:** Average silent site divergence (*K_s_*) and estimated age of split (bold) between *M. truncatula *and related species from the vicioide subclade, using three chloroplast gene regions: *rbcL, matK *and *trnL*

	*Melilotus*	*Trifolium*	*Pisum*	*Vicia*
*Medicago truncatula*		***rbcL***		
	
	(0.0590) **9.1 My** *M. albus*	(0.0882) **13.6 My** *T. pratense*	(0.1194) **18.5 My** *P. sativum*	(0.1556) **24.1 My** *V. cracca*
	
		***matK***		
	
	(0.0511) **6.7 My** *M. albus*	(0.0661) **8.7 My** *T. albopurpureum*	(0.1149) **15.2 My** *P. sativum*	(0.1103) **14.6 My** *V. hirsuta*
	
		***trnL***		
	
	(0.0126) **6.6 My** *M. albus*	(0.0254) **13.2 My** *T. praetermissum*	(0.0211) **11.0 My** *P. sativum*	(0.0297) **15.5 My** *V. nipponica*

Average Age of split	**7.5 My**	**11.8 My**	**14.9 My**	**18.1 My**

As a calibration point we used the 34 million years estimate for the split between *Pisum *and *Albizia *species [27] and the levels of synonymous divergence per synonymous site (*K_s_*) for the published chloroplast genes *rbcL*, *matK *and *trnL *of *Pisum sativum *and three *Albizia *species, namely the *K_s _*values of 0.2199 for *P. sativum/A. julibrissin; *0.2575 for *P. sativum/A. versicolor *and 0.0653 for *P. sativum/A. julibrissin*, respectively.

To test the presence of the *GS2b *gene in *Melilotus *species, primers have been designed for conserved regions in the coding sequences of GS2 genes from *L. japonicas*, *P. sativum*, *Phaseolus vulgaris*, *Glycine max *and the two *M. truncatula *GS2 genes (see Material and Methods). The ~ 960 bp amplification product obtained using these primers and genomic DNA of *Melilotus albus *was cloned. Two restriction patterns, which were called M. albus 1 and M. albus 2, were revealed from the analysis of 36 colonies using two restriction enzymes. Sequencing results revealed that M. albus 2 represent two types of sequences, called M. albus 2-1 and M. albus 2-2. Using blastn the three sequences revealed more than 95% similarity with *M. truncatula MtGS2a *coding region (AY225150) and less than 75% similarity with the *MtGS1 *genes. Therefore, the putative coding region of these sequences was annotated according to the *Medicago *sequence. In these sequences there are five putative introns in the region analysed, with intron sizes similar to those observed in *Medicago *(AC202342). Although the specific function of the *Melilotus albus *GS2b genes can not be inferred from these studies, the region analysed suggests that they may be functional.

**Figure 3:**
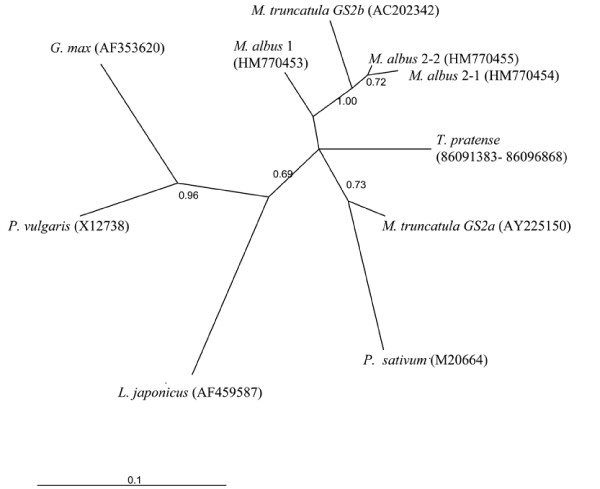
**Phylogenetic relationships of GS2 genes in relevant legume species**. Bayesian unconstrained tree. In brackets are GenBank accession numbers. Numbers represent posterior probability values.

The phylogenetic relationship of the *Melilotus albus *sequences and *M. truncatula GS2 *genes, using the coding region is presented in Figure 3. It should be noted that M. albus 2-1 and M. albus 2-2 sequences cluster with *M. truncatula MtGS2b*, with a strong bootstrap support. Diversity levels between M. albus 2-1 and M. albus 2-2 at the coding region are *K_s _*= 0.0142 and *K_a _*= 0.0042. Therefore, these sequences may represent two different *GS2b *genes in *Melilotus albus*, from a very recent (about 1 MY old) duplication. M. albus 1 sequence does not cluster with strong support with both *MtGS2a *and *MtGS2b *gene sequences. To address if this sequence may represent the orthologue of *M. truncatula MtGS2a *gene the Bayesian tree presented in Figure 3 was constrained on having M. albus 2-1 and M. albus 2-2 and *M truncatula MtGS2b *sequences as one group, plus all other sequences as another group. When the difference of Bayes factors between this tree and the unconstrained tree is calculated, a value of 4.85 is obtained. It has been suggested that the constrained tree should be considered as significantly worse, only if twice this difference gives a number higher than 10 [30]. Thus, the constrained tree is not significantly worse than the unconstrained one, and M. albus 1 sequence may represent the orthologue of *M. truncatula MtGS2a *gene.

### Isolation and characterization of two *MtGS2b *transcripts

To investigate whether *MtGS2b *is a functional gene, a thorough search for *MtGS2b *transcripts was performed in several organs of *M. truncatula *by RT-PCR. The high homology between the coding sequences of *MtGS2b *and the previously characterized *MtGS2a*, prevented the design of *MtGS2b *specific primers or probes. As an alternative approach, a set of primers that amplify both transcripts was used and the two transcripts were subsequently differentiated by restriction digestion of the PCR product by an enzyme that differentially cuts *MtGS2b*. This first approach pointed to a specific expression of *MtGS2b *in developing seeds (Data not shown). This tissue was thus used to isolate and sequence the 5'and 3' ends of *MtGS2b *mRNAs by RACE. Gene specific primers were designed for the poorly conserved 5' UTR allowing subsequent cloning of *MtGS2b *cDNAs. Interestingly, two different *MtGS2b *transcripts of 1774 bp and 1624 bp were isolated and designated as *MtGS2b-α *and *MtGS2b-β*, respectively. The two transcripts were sequenced and found to be identical in their coding sequence and 3'UTR, but different in the length and sequence of the 5'UTR (245 bp for *MtGS2b-α *and 95 bp for *MtGS2b-β)*. Sequence alignment of the two cDNAs with the genomic DNA indicates that the two transcripts arise from alternative splicing over the first intron. *MtGS2b-α *results from the retention of the first intron (Figure 2). As the position of the start codon is not altered, the two transcripts are predicted to encode a similar protein. The two *MtGS2b *mRNA sequences have been posted in GenBank, accession numbers HM775420 and HM775421 for *MtGS2b-α *and *MtGS2b-β*, respectively.

### Expression analysis of *MtGS2b *transcripts and proteins

**Figure 4:**
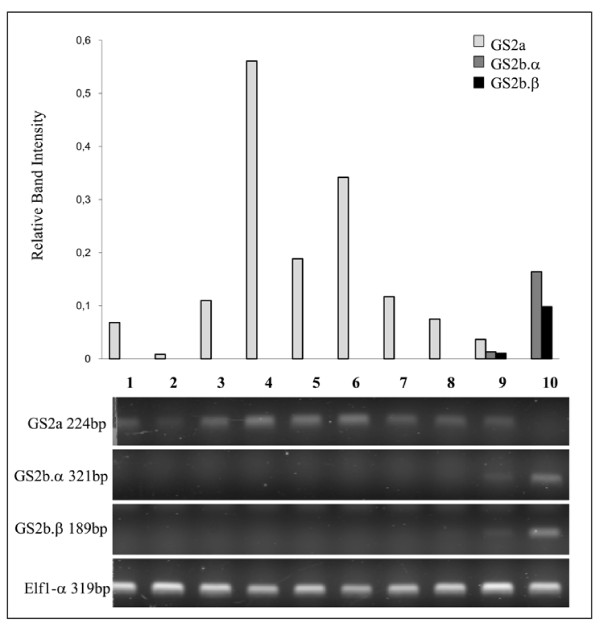
**Semi-quantitative RT-PCR analysis of GS2 trancripts in different organs of the plant**. Total RNA was extracted from roots of plants growing on ammonium nitrate (1), roots of plants growing in symbiosis with *S. meliloti *(2), 14 day old nodules (3), leaves (4), stems (5), cotyledons from light grown seedlings (6), cotyledons from dark grown seedlings (7), flowers (8), pods (9) and developing seeds collected at 20 DAP (10). 50 ng of cDNA was used in each assay. Transcript levels for *MtGS2a*, *MtGS2b-α, MtGS2b-β *and the elongation factor 1-a (*elf1-a*), as internal reference gene are shown. Values correspond to quantified band intensities of GS2 relative to the housekeeping gene elf1-α.

**Figure 5:**
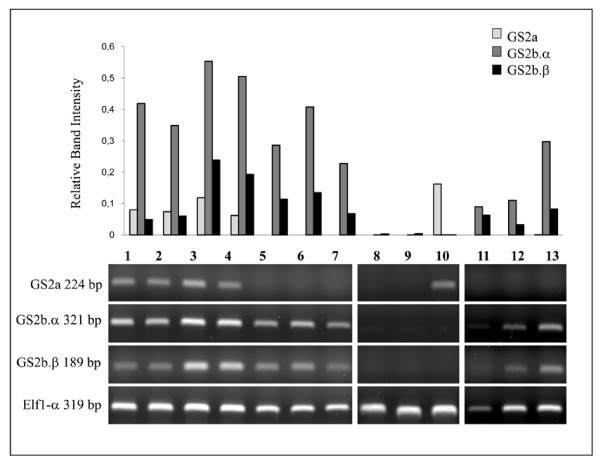
**Semi-quantitative RT-PCR analysis of GS2 transcripts during seed development, seed imbibition and in major seed tissues**. Total RNA was extracted from 3 DAP seeds (1), 6 DAP (2), 10 DAP (3), 14 DAP (4), 20 DAP (5), 24 DAP (6) and 36 DAP (7), dry seeds (after-ripenning) (8), dry seeds 1,5 h after imbibition (9) and dry seeds 24 h after imbibition (10), seed coat (11), endosperm (12) and whole embryo (13). 50 ng of cDNA was used in each assay. Transcript levels for *MtGS2a*, *MtGS2b-α, MtGS2b-β *and the elongation factor 1-a (*elf1-α*), as internal reference gene are shown. Values correspond to quantified band intensities of each GS2 transcript relative to the housekeeping gene elf1-α.

The expression of *MtGS2a *and *MtGS2b *was evaluated by semi-quantitative RT-PCR using specific primers. To be able to distinguish *MtGS2a *and the two *MtGS2b *alternatively spliced transcripts, forward primers were designed to hybridize specifically in the non-conserved 5'UTRs. These primers were used to amplify total RNA isolated from several organs of the plant. The results are displayed as gel images and graphical representation of the amount of RNA relative to the housekeeping gene *elf1-α *(TC106485) (Figures 4 and 5).

*MtGS2a *transcripts were detected in almost all organs of the plant, being generally more highly expressed in green tissues (leaves, stems and cotyledons from light-grown seedlings) and poorly expressed in non-green tissues. Interestingly and despite the nearly ubiquitous pattern of expression in green tissues, *MtGS2a *mRNAs could not be detect in developing, green seeds (Figure 4, lane 10), contrasting with the expression of both *MtGS2b *transcripts exclusively in these tissues. The *MtGS2b *expression signal detected in pods (Figure 4, lane 9) is attributed to the green seeds contained inside the pod, as asserted by independent RT-PCR analyses of pod walls separated from green seeds (data not shown).

The seed-specific nature of *MtGS2b *expression demanded an evaluation of its expression during seed development and in dissected seed tissues (Figure 5). Semi-quantitative RT-PCR was performed on total RNA extracted from seeds collected at seven key developmental stages previously characterized at the physiological level [3]: 3 days after pollination (DAP) (end of seed coat differentiation), 6 DAP (globular stage embryo), 10 DAP (early torpedo embryo), 14 DAP (early stages of seed filling), 20 DAP (peak of accumulation of storage compounds) 24 DAP (late stages of seed filling) and 36 DAP (stage of physiological maturity and desiccation) and from embryos, seed coat and endosperm dissected from 20 DAP seeds. *MtGS2a *transcript levels remain roughly unaltered from 3 DAP to 14 DAP (Figure 5, lanes 1 to 4) however, at 20 DAP and thereafter, it seems to be absent (Figure 5, lanes 5 to 7) from developing seeds. In contrast, the two *MtGS2b *transcripts were detected in all developmental stages. The overall pattern of expression appears to be similar for the two *MtGS2b *alternatively spliced transcripts, with *MtGS2b-α *always being expressed at higher levels. Although both transcripts seem to reach a maximum of accumulation at 10 DAP (Figure 5, lane 3), there was not a clear tendency to either decline or increase which may indicate that the levels are somehow constant during seed development. The tissue distribution of *MtGS2b *transcripts was also evaluated using dissected tissues from seeds collected at 20 DAP: seed coat, endosperm and embryo (Figure 5, lanes 11 to 13). Both *MtGS2b *transcripts were found to be present in the three major seed tissues with higher expression levels in the embryo. Thus, from the two *M. truncatula *GS2 encoding genes, *MtGS2b *seems to be the most significant GS2 gene expressed in developing seeds, and the only GS2 gene expressed by the end of seed-filling and late maturation stages

To evaluate whether *MtGS2b *expression could be related to seed germination, the characterization of GS2 gene expression was extended to after-ripened seeds and to seeds after imbibition. Both GS2 transcripts were detected only at basal levels in mature dry seeds and in seeds 1.5 hours following imbibition (Figure 5, lanes 8-9). However 24 hours after seed imbibition, when the radicle was already visible, *MtGS2b *expression was undetectable. At this stage, a substantial increase in *MtGS2a *expression was observed (Figure 5, lane 10) as previously reported to occur in *M. truncatula *during post-germinative growth [31].

**Figure 6:**
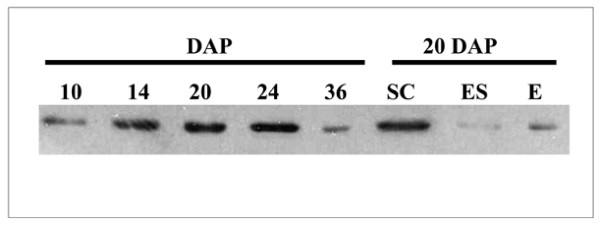
**Western blot analysis of GS2 polypeptides during seed development and in major seed tissues**. Proteins were extracted from seeds collected at different times following pollination (as indicated) and in seed tissues dissected from 20 DAP seeds: seed coat (SC), endosperm (ES) and embryo (E). Protein extracts (10 μg per lane) were subjected to SDS-PAGE followed by western-blot analysis using a specific anti-GS2 antibody.

To correlate GS2 transcript levels with GS2 polypeptide content in developing seeds, protein extracts from five of the key developmental stages (10 DAP to 36 DAP) and the three major dissected seed tissues (seed coat, endosperm and embryo), were subjected to SDS-PAGE followed by western blotting using a specific GS2 antibody. The small size of 3 DAP and 6 DAP seeds disallowed the collection of sufficient material to include in this analysis. GS2 polypeptide levels seem to increase during the course of seed development until the end of the seed-filling stage (24 DAP) with a significant decrease in abundance at 36 DAP (Figure 6). Although the anti-GS2 antibody cannot differentiate between the products of *MtGS2a *and *MtGS2b*, the polypeptides detected at 20 DAP and afterwards are likely to result from *MtGS2b *expression, since *MtGS2a *transcripts are undetectable at these stages of development. GS2 was mostly abundant in the seed coat, however the embryo and the endosperm also contribute to the GS2 polypeptide content in 20 DAP seeds (Figure 6). These results do not entirely reflect the transcription profile of the genes, indicating the existence of regulatory controls operating at a post-transcriptional level.

### Evaluation of the functionality of *MtGS2b *isoenzymes

**Figure 7:**
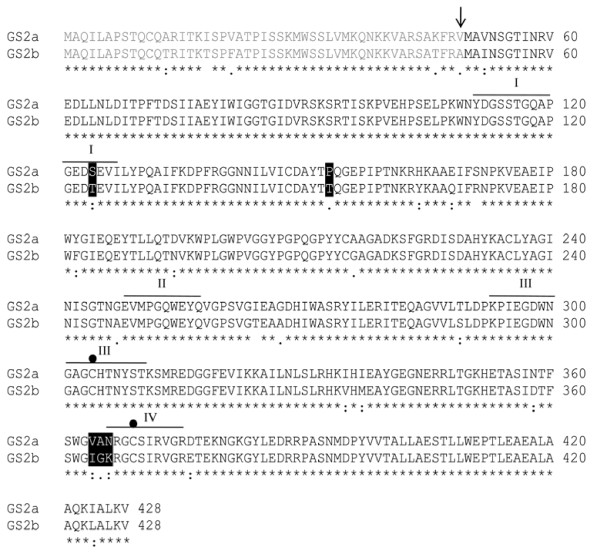
**Clustal X alignment of GS2a and GS2b deduced amino acid sequences**. Residues fully conserved (*), highly conserved (:), poorly conserved (.) and not conserved (). The transit peptide is represented in gray and was predicted by analogy with the GS2 from pea [9]. Black boxes outline potential modified residues affecting GS2b activity. The black dots indicate two cysteine residues which are conserved in all plastid located isoenzymes [69]. I-IV-identifies the conserved segments between GS proteins defined by Eisenberg et al. [49].

*MtGS2b *contains the information to encode a functional protein quite similar to GS2a. A comparison of the deduced amino acid sequences of the two *M. truncatula *GS2 polypeptides, reveals a high degree of homology (94% amino acid identity), with only 26 amino acid substitutions in a total of 428 residues (Figure 7). Five of the amino acid substitutions are located in the N-terminal transit peptide and thus the mature protein is predicted to contain only 21 amino acid substitutions. GS2a and GS2b mature proteins are expected to have similar molecular masses, but different pIs, of 5.25 and 5.43, respectively. Based on structural analysis [32,33], five amino acid substitutions are predicted to be in positions that could affect GS activity.

**Figure 8:**
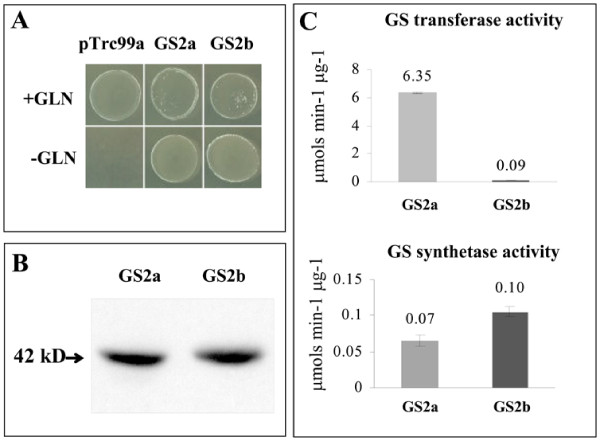
**Analysis of the catalytic functionality of GS2b**. A. Complementation analysis of an *E. coli glnA *mutant expressing either GS2b or GS2a plant cDNAs. The plant cDNA corresponding to the coding sequences, without the predicted transit peptide were introduced into pTrc99A. Controls were transformed with the empty plasmid. Bacteria were grown on Minimal Medium (M9) agar plates with (+GLN) and without (-GLN) a glutamine supplement (0.25 mg mL-1). B. Western blot analysis of *E. coli glnA *extracts expressing recombinant GS2a or GS2b mature proteins probed with anti-GS antibody. C. GS activity assays of *E.coli glnA- *extracts expressing either GS2a or GS2b mature proteins. Results represent the mean of three independent experiments.

To ensure that the protein encoded by the newly identified *MtGS2b *gene is catalytically functional, heterologous complementation assays were performed. The plant *MtGS2b *cDNA was cloned, without the target peptide, into the *E. coli *expression vector pTrc99A, and introduced into the the *E*. *coli glnA *mutant strain ET8894. The previously described GS2a recombinant protein [12] was used as positive control and the empty plasmid as a negative control. Complementation, enabling growth on minimal media containing ammonium as the nitrogen source, was clearly observed for the two GS2 constructs whereas the bacteria transformed with the vector alone, could only grow in the presence of a glutamine supplement (Figure 8A), indicating that GS2b is catalytically and physiologically active.

Western blot analysis of total soluble protein extracts from the two different bacterial cultures (Figure 8B) showed that the polypeptides are expressed in the bacterial host with the expected molecular mass of approximately 42 kDa. To compare the activity of the two proteins, GS activity assays were performed on total proteins extracted from the *glnA- *strain grown in liquid M9 minimal medium plus IPTG. GS activity determined by the transferase reaction was 70 times lower for GS2b relative to GS2a, while the synthetase activity was 1.3 fold enhanced (Figure 8C). This reflects striking differences in the kinetic properties of GS2b. Several attempts were performed to express the protein GS2b with a 6×His tag in *E. coli *in order to purify the enzyme and determine kinetic parameters. All attempts were unsuccessful, despite the fact that the highly homologous GS2a can be easily expressed [34], preventing further kinetic characterization of the enzyme.

## Discussion

*M. truncatula *is the first plant species in which a second GS2 gene has been detected. The gene is located on the same chromosome (chromosome 2) as the previously characterized *MtGS2a *gene, only 8 Kb apart, indicating a recent duplication event. Gene duplicates provide an opportunity for functional innovation. Mutations or genomic rearrangements altering when and where the duplicates are expressed, or the structure/function of the products encoded by the genes, can provide a selective advantage to the organism and be subsequently retained [35]. The newly identified *MtGS2b *gene, shows a seed specific expression and may represent a functional innovation of plastid located GS related to legume seed metabolism. This idea is reinforced by the presence of a second GS2 gene in a closely related species of the *Vicioide *subclade (*Melilotus albus*). Phylogenetic analysis estimated the age of the duplication event to have occurred 10 My ago, after legume speciation.

GS genes have been the subject of many molecular evolution studies, the gene is considered one of the oldest functioning genes [36], and a good molecular clock [37-39]. Phylogenetic studies have shown that chloroplastic and cytosolic GS genes have evolved through gene duplication. Kumada et al. [36] estimated the common ancestor of the two classes of genes to have duplicated about 300 My ago, before the monocot/dicot divergence. Doyle [38] proposed that members of the cytosolic GS gene family in plants have also evolved by duplication of a single, ancestral cytosolic GS gene and the evolution of separate gene copies to fulfil different metabolic cell requirements. A similar evolutionary process is conceivable for the plastid GS genes, the newly identified *MtGS2b *gene could have evolved to fulfil a specific metabolic role related to legume seed metabolism. To our knowledge, this is the first report of a duplication of a plastid located gene, and interestingly, the duplication seems to be quite recent in view of the estimation of the age of divergence of the cytosolic GS genes. Studies in *Pinus sylvestris*, estimate that the duplication of an ancestral cytosolic GS occurred long before the divergence of gymnosperms/angiosperms [40].

An exceptional example of gene conversion has been proposed for the origin of the *P. sativum *cytosolic GS3A and GS3b twin genes [41]. The coding regions of plant GS genes are generally well conserved whereas the noncoding sequences are often very divergent, but the non-coding regions (5' and 3' UTR and introns) of *P. sativum *cytosolic GS3A and GS3B twin genes are highly conserved. These two genes are located on separate *loci *in different chromosomes and gene conversion has been invoked as a possible mechanism for maintaining the high level of nucleotide similarity between the twin genes [41]. Although the *Medicago truncatula MtGS2a *and *MtGS2b *genes show a high degree of sequence conservation in both the coding and non-coding regions (see Results), the two genes are located in the same chromosome only 8 kb apart, compatible with an adjacent duplication event.

A surprising feature of *MtGS2b *expression is the fact that it is subjected to alternative splicing. Alignment of the mRNA sequences with the genomic sequence indicates that the alternative splicing occurs over the first intron, resulting in a longer 5' UTR of alternatively spliced form α by retention of the first intron. Intron retention is considered the prevalent mode of alternative splicing in plants [42,43]. In the case of *MtGS2b*, intron retention occurs on the 5' UTR and does not affect the protein identity, but seems to be related to mRNA stability and/or translation efficiency. Leader introns have been reported to increase transcription and/or translation efficiencies of some plant genes. Curi et al. [44] demonstrated that the leader intron of a gene encoding cytochrome c oxidase in Arabidopsis is responsible for increasing translation efficiency, besides being also responsible for the tissue-specific expression of the gene. Another example is the 5'UTR intron of rice polyubiquitin, which was found to be responsible for transcriptional, post-transcriptional enhancement and moderate increase in translation [45]. The two alternatively spliced *MtGS2b *transcripts show essentially the same pattern of expression during seed development (Figure 5), with the *MtGS2b-α *transcripts always being more abundant than those of *MtGS2b-β*. This suggests that either the excision of the first intron is inefficient, or that mechanisms exist so that *MtGS2b-α *mRNA levels are maintained.

On the other hand, the steady state levels of the two *MtGS2b *mRNAs do not always correlate with protein abundance. Similarly, a recent study which compared the proteome and transcriptome of *M.truncatula *seeds revealed several proteins displaying a profile differing from that of the corresponding transcripts during seed development and in different seed tissues [3]. The abundance of *MtGS2b-α *transcripts seems to be inversely proportional to GS2 polypeptide abundance, most notorious at 10 and 14 DAP, suggesting that the *MtGS2b-α *additional 5'UTR intron functions to reduce mRNA translation, as shown to occur for the chick proinsulin gene, where retention of a 5'UTR intron leads to nearly complete translation blocking without affecting mRNA stability or transport [46]. To our knowledge, this is the first report of regulation of a GS gene by alternative splicing by intron retention in the 5' UTR, but it is becoming increasingly evident that 5' introns can have a dramatic effect on gene expression in plants [47,48].

The seed specific expression of the gene, strongly suggests a role related to seed metabolism. Here, evidence is presented that the enzyme produced by *MtGS2b *is catalytically and physiologically active as it was able to rescue the phenotype of an *E. coli glnA *mutant (Figure 8), but GS2b enzymatic activity presents distinctive features in relation to its homologue GS2a. Although, we were unable to purify the protein, due to difficulties of expression in *E. coli*, activity tests performed on crude extracts, revealed significant differences in the kinetic activity of the two enzymes. The synthetase: transferase activity ratio of the new enzyme GS2b is surprisingly increased in relation to all the other *Medicago truncatula *GS isoenzymes (cytosolic or plastidic) [20]. Besides its biosynthetic reaction, GS catalyzes the hydrolysis of glutamine into glutamate and ammonia in the presence of the cofactors ADP and Pi. This reaction may be important in the production of ammonia and in providing glutamate for specific metabolic proposes. The unique synthetase to transferase GS activity ratio of this new GS2 isoenzyme, together with its seed specific expression is compatible with a specific function of the enzyme under a defined metabolic context exclusive to seed metabolism.

The difference in kinetic activity of the two *M. truncatula *GS2 isoenzymes is surprising in view of the high sequence similarity between the two GS polypeptides (94% aminoacid identity). However there are 26 differences between the two *M. truncatula *amino acid sequences with 11 of these being non-conservative. Based on structural data [32,33] it is predicted that most of the amino acid substitutions are not in positions critical for GS activity (Figure 6) except for residues I364, G365 and K366. These three sequential residues are particularly interesting since K366 integrates the GS conserved region IV defined by Eisenberg et al. [49] and known to participate in the active site. These three amino acid substitutions could be responsible for the particular GS synthetase to transferase ratio of GS2b.

At the initial stages of seed development both *M. truncatula *GS2 genes are simultaneously expressed, raising the question of whether *MtGS2a *and *MtGS2b *perform redundant roles in the seed or if *MtGS2b *acquired novel biochemical functions following gene duplication. The expression pattern of the two genes during seed development favours the second hypothesis. *MtGS2b *was found to be exclusively and continuously transcribed during seed development, whereas the expression of *MtGS2a *was down regulated at the onset of storage compound accumulation and reappears during post-germinative growth (Figure 5). The stronger and specific expression of *MtGS2b *during seed filling, strongly suggests that the enzyme performs a seed specific role related to reserve accumulation. In leaves, the main function of plastidic GS is the reassimilation of ammonia resulting from photorespiration [7,8]. It is unlikely that the enzyme performs a similar function in the seed, because seeds have low Rubisco activity [50] and low internal O_2 _levels, two conditions required for photorespiration to occur [51]. In roots, GS2 is mainly implicated in assimilating the ammonia derived from nitrate reduction [52]. This function is also unlikely for the seed GS2, since the primary source of nitrogen available to the developing seeds is amino acids, mainly glutamine and asparagine [53]. A possible function for a plastid-located GS in legume seeds, and particularly during seed filling, would be N-remobilization from the amide amino acids, asparagine and glutamine to other amino acids required for storage-protein synthesis. The breakdown of asparagine is catalysed by asparaginase, which releases ammonia for reasssimilation via GS.

During the filling stage, seeds accumulate proteins, oils and carbohydrates to support seed germination and post-germinative growth [54]. *M. truncatula *produces protein-rich seeds (40%) with high lipid (10% fatty acids) and low starch content (< 1%) [55]. Some of these storage compounds are accumulated in the seed plastids, which perform specific metabolic features and undergo deep changes during seed development. *M. truncatula *possesses green embryos, and during seed development, the chloroplasts gradually differentiate into storage plastids [56]. Photosynthesis has a low input to the overall carbon synthesis and seems to be mainly directed to oxygen and ATP production to support the biosynthesis of storage compounds [57-59]. The plastid differentiation involves chlorophyll and photosynthetic machinery degradation and a plastidic glutamine synthetase could also be involved in the remobilization of nitrogen released during the degradation of the photosynthetic machinery.

The importance of seed plastid metabolism in the partitioning of assimilates into seed storage compounds, has been recently evidenced in several legumes. In *Vicia narbonensis*, the antisense inhibition of the Glucose-6-phosphate/phosphate translocator, an importer of carbon to plastids, was found to reduce the levels of starch but to increase storage protein biosynthesis [60]. More recently, a pea 2-oxoglutarate/malate translocator (OMT) was also found to have a crucial role during seed storage. OMT repression leads to reduced conversion of carbohydrates from sucrose to amino acids and proteins and delayed storage plastid differentiation. The authors emphasize that one of the probable causes of the phenotype displayed is the substrate limitation of plastidial GS/GOGAT cycle [61].

## Conclusions

In conclusion, this study shows that *Medicago truncatula *contains an additional GS gene encoding a plastid located enzyme, which is functional and exclusively expressed during seed development. GS2 gene duplicates exist in a closely related species and it is estimated that the duplication event occurred around 10 My ago. The seed specific pattern of expression, together with the kinetic differences of this new isoenzyme in relation to the previously characterized *MtGS2a*, strongly suggests that the enzyme is performing a seed specific role related to storage protein accumulation. It is conceivable that the unique metabolism of legume seed plastids supplied the selective pressure for the unique expression and kinetic properties of this novel plastid located GS, in order to more efficiently support storage compound biosynthesis in *M. truncatula*.

## Methods

### Plant material and growth conditions

Plants of *Medicago truncatula *Gaertn. cv Jemalong and *Melilotus albus *were grown under 16 h light (22°C)/8 h dark (19°C) cycles under a light intensity of 150-200 μmol m^-2 ^s^-1^. Roots, leaves, and stems were collected from five week old plants grown aeroponically on ammonium nitrate in the medium described by Lullien et al. [62]. Cotyledons were collected from seedlings either kept in the dark for two days or exposed to light. For nodule induction, plants were nitrogen starved for seven days before inoculation with the *Sinorhizobium meliloti *strain Rm1021 pXLGD4 RCR 2011(GMI51) and root nodules were collected 14 days after inoculation. For flower, pod and seed collection, plants were grown on soil and fertilized once a week. For seed development studies, individual flowers were tagged on the day of flower appearance and seeds were collected from pods harvested at different time points from 3 to 36 days after pollination (DAP) according to Gallardo et al. [3]. Seed coat, endosperm and embryo were separated from seeds collected 20 DAP and dissected under a Stereo Microscope. Finally, for seed imbibition studies, dry seeds were surface scarified/sterilized and frozen immediately or after 1.5 and 24 hours of water imbibition. All biological material was immediately frozen in liquid nitrogen and stored at -80°C.

### Nucleic acid extraction

Genomic DNA was extracted and purified from young leaves of *M. truncatula *or *M. album*, and from BAC DNA clone mth2-53e90 essentially as described in Sambrook et al.[63]. Total RNA was extracted from 100 mg of plant tissue, using the RNeasy Plant mini Kit (Qiagen) according to the manufacturer's instructions. All Nucleic acids were quantified using a Nanodrop spectrophotometer (Thermo scientific).

### Southern blot analysis

20 μg of genomic *M. truncatula *DNA and 5μg of BAC mth2-53e90 DNA were digested with *EcoRV*, *NcoI *and *BglII *(New England Biolabs, UK). DNA fragments were separated on a 0.8% agarose gel and blotted onto Amersham Hybond™-N^+ ^Nylon membranes (GE healthcare). The blotted membrane was hybridized with a *MtGS2a *(GenBank: AY225150.1) specific probe, corresponding to 260 bp of the 5' coding sequence (CDS). Probe labeling using the DIG DNA labeling Kit (Roche, Applied Science) and hybridization were performed under high stringency conditions, according to the manufacturer's instructions.

### Sequence analysis

*MtGS2b *gene identification was achieved through Blast Database searches using NCBI server http://blast.ncbi.nlm.nih.gov/Blast.cgi and the *M. truncatula *sequencing resources http://www.medicago.org/genome/. Organization of both GS2 genes and prediction of *MtGS2b *coding sequence was carried out using the FGENESH2.6 software from Softberry http://www.softberry.com. *MtGS2a *and *MtGS2b *amino acid sequences were deduced from the cDNAs and aligned using Clustal X software [64].

### Estimation of the age of split between four species of the Vicioide subclade and *M. truncatula*

To estimate the age of separation of four species of the vicioide subclade of the IRLC (the inverted-repeat-lacking) clade (see figure five in [28]) from *M. truncatula*, three genes for which nucleotide sequence data are available for at least one species of *Melilotus*, *Trifolium*, *Pisum*, *Vicia*, and *Albizia *were used, namely *rbcL*, *matK *genes and the intron region of *trnL *(accession numbers are in additional file 1, Table S1). The nucleotide sequences were aligned using ClustalX [64]. Levels of synonymous divergence per synonymous site (*K_s _*values with Jukes-Cantor correction) that, under the assumption of a molecular clock, are proportional to time, were computed using the DNasp software [65]. As a calibration point we used the 34 million years estimate for the split between *Pisum *and *Albizia *species [27].

### Estimation of the age of the *M. truncatula *GS2 duplication event and amplification of GS2 genes in species inferred to have the gene duplication

To estimate the age of the *M. truncatula GS2 *gene duplication event, the methodology described in the previous section was used for the following sequences: *M. truncatula MtGS2a *and *MtGS2b *and *Pisum sativum GS2 *(for calibration purposes). The estimated age of 14.9 My for the separation between *Pisum *and *Medicago *was used to estimate the age of the *M. truncatula GS2 *gene duplication. The comparison of the estimated age for the duplication event with the ages for the separation of species of the vicioide subclade from *M. truncatula *(see previous section) revealed that the gene duplication likely occurred before the separation of the *Medicago *and *Melilotus *genera.

Genomic DNA from *Melilotus albus *was amplified using primers Gs2F1 and Gs2R1 (additional file 2 Table S1), designed to align with conserved regions of the coding sequence of the *L. japonicus *(AF459587), *P. sativum *(M20664), *M. truncatula MtGS2a *and *MtGS2b *(AY225150; AC202342), *Phaseolus vulgaris *(X12738), and *Glycine max *(AF353620). Standard amplification conditions were 35 cycles with denaturation at 94°C for 30 seconds, primer annealing at 50°C for 30 s, and primer extension at 72°C for 3 min. As these primers supported the amplification of the two *GS2 *genes in *M. truncatula*, the *Melilotus albus *amplification product of ~950 bp was cloned. This amplification product is expected to be the result of the amplification of both *M. albus GS2 *genes. Therefore, for 36 colonies the amplification product of ~500 bp obtained with primers GS2F2 and GS2R2 (amplification conditions as described above) were digested with *AluI *and *Sau3A*. For each restriction fragment three colonies were sequenced in order to obtain a consensus sequence. The ABI PRISM BigDye cycle-sequencing kit (Perkin Elmer, Foster City, CA), and specific primers, or the primers for the M13 forward and reverse priming sites of the pCR2.1 vector, were used to prepare the sequencing reactions. Sequencing was performed by STABVIDA (Lisboa, Portugal). The *Trifolium pratense *sequence included in the phylogeny was obtained from the consensus of the 46 EST sequences, out of 38109 available for this species, retrieved using BLASTn and the *M. truncatula MtGS2a *sequence as a query. It should be noted that for the region considered these sequences are identical. Alignment was performed as described above. For Bayesian tree estimation we used MrBayes [66] with the generalized time reversible (GTR) model of sequence evolution, allowing for among site rate variation and a proportion of invariable sites. Third codon positions were allowed to have a gamma-distributed rate with a different shape parameter than the first and second positions. Two simultaneous, completely independent analyses starting from different random trees were run for one million generations (each comprising one cold and three heated chains). Samples were taken every 100^th ^generations. The first 2500 samples were discarded (burnin).

### RACE and *MtGS2b *cDNA isolation

Rapid amplification of 5' and 3' cDNA ends was carried out using FirstChoice^® ^RLM-RACE Kit (Ambion^®^) according to the manufacturer's instructions, with some minor adaptations. The 5'RLM-RACE first-strand cDNA synthesis used 6 μg of total RNA while 3'RACE used 1μg. Both 5' and 3' primary (outer) and nested (inner) PCRs were performed using *Pfu *DNA polymerase (Fermentas, Life Sciences). Primers were designed according to the coding sequence that was deduced from the genomic sequence (additional file 2 Table S2). The resulting PCR fragments were cloned into pCR^® ^Blunt Vector (Invitrogen, Life Science) and individual clones were sequenced. Gene specific primers were designed to anneal at the beginning of the 5'UTR and at the end of 3'UTR. PCR amplification using these primers, *Pfu *DNA polymerase (Fermentas, Life Sciences) and 50 ng of first-strand cDNA resulted in the isolation of two PCR fragments of approximately 1700 bp (GS2b-α) and 1600 bp (GS2b-β) that were further cloned into pCR^® ^Blunt Vector (pCR^®^Blunt-GS2b constructs). All sequencing results were obtained using an automatic capillary sequencer (Stab Vida, Portugal). RACE-PCRs and the isolation of *MtGS2b *full-length cDNAs were performed on total RNA from a pool of differently developed seeds. The primer sequences are indicated in additional file 2, Table S2.

### RT-PCR Analysis

For RT-PCR, 5 μg of total RNA were reverse transcribed using Superscript-RT™ III (Invitrogen, Life Science) and Random Hexamers according to the manufacturer's instructions. Semi-quantification of *MtGS2b-α*, *MtGS2b-β*, *MtGS2a *and elongation factor 1-α (*Elf1-α*) transcript levels was performed using a reaction mix composed of 50 ng of cDNA as template, 2 mM dNTP's, 10 pmol primers (additional file 2, Table S3), 1.5 mM Mg^2+^, and 0.25 units of Taq DNA Polymerase (AbGene Limited) in a total volume of 50 μL. The amplification conditions were as follows: (1×) 95°C, 2 min; (30×) 95°C, 30 s; each primer pair amplification temperature, 30 s; 72°C, 30 s. The amplified fragments were separated on 2% agarose gels and labeled with SYBR^® ^Safe DNA Stain (Invitrogen, Life Science). Gel images were obtained using a Typhoon 8600 variable mode Imager (GE Healthcare, Lifesciences) and band intensities were quantified using the ImageQuant 5.1 software (Molecular Dynamics).

### Expression of *MtGS2b *recombinant protein in *Escherichia coli*

*MtGS2b *coding sequence for the predicted mature protein, that is, without the sequence corresponding to the transit peptide, was amplified from the pCR^®^Blunt-GS2b constructs with Platinum^® ^Taq High Fidelity enzyme (Invitrogen, Life Science) using *MtGS2b *specific primer (5'GCAATCAACTCTGGCACC3') and M13 foward primer. The resulting 1416 bp PCR amplified fragment was further digested with *SalI *(New England Biolabs) and inserted in frame in the *NcoI/XhoI *sites of pTrc99A vector (GE healthcare, Lifesciences), with the *NcoI *site as a blunt end. The resulting construct pTrc99a-GS2b was transformed into the *Escherichia coli glnA *mutant ET8894 [67]. The cloning strategy used for *MtGS2a *is described in Melo et al. [12] as well as the procedures for the complementation assays.

### Preparation of soluble protein extracts from *E. Coli *and plant tissues and GS Activity Assays

Plant material and bacterial pellets were homogenized at 0°C to 4°C using a mortar and pestle as described before [12]. GS activity was determined using both the transferase and biosynthetic assays [68]. One unit of activity is equivalent to 1μmol min ^-1 ^μg^-1 ^glutamyl hydroxamate produced at 30°C.

### Western Immunoblotting

Soluble protein concentration was measured by the Coomassie dye-binding assay (Bio Rad) using BSA as a standard. 10 μg of total soluble protein extracts were separated by 12.5% (w/v) SDS-polyacryamide gel electrophoresis (SDS-PAGE) and electroblotted onto nitrocellulose membranes (Schleicher & Schuell). Immunodetection was performed with a rabbit polyclonal anti-peptide (SKSRTISKPVEHPSEL) antibody (Eurogentec), synthesized to specifically detect plastid-located GS isoenzymes and does not recognize cytosolic GS isoenzymes. Immunodetection was performed using goat anti-rabbit peroxidase conjugated antibody (Vector Laboratories) and ECL™ (GE healthcare, Lifesciences) detection system.

## Authors' contributions

ARS carried out all the experimental work, participated in the design of the study and helped to draft the manuscript. CPV carried out the molecular phylogenetic analysis and drafted the corresponding text. JVC participated in the design of the study and helped to draft the manuscript. HGC conceived and coordinated the study and drafted the manuscript. All authors read and approved the final manuscript.

## Supplementary Material

Additional file 1 **GenBank accession numbers of DNA sequences used for phylogenetic analysis**. GenBank accession numbers for three chloroplast gene regions used to estimate the age of separation of different species of the vicioide subclade. Click here for file

Additional file 2 **List of primers**. Sequence of the primers used to amplify *GS2 *genes from *Medicago truncatula *and *Melilotus alba*. Click here for file
