# Supplementary material for: Medicago truncatula contains a second gene encoding a plastid located glutamine synthetase exclusively expressed in developing seeds
Source: BMC Plant Biol. 2010 Aug 19;10:183. doi: 10.1186/1471-2229-10-183 (PMC3095313; doi:10.1186/1471-2229-10-183)
Supplement: Additional file 1 — GenBank accession numbers of DNA sequences used for phylogenetic analysis. GenBank accession numbers for three chloroplast gene regions used to estimate the age of separation of different species of the vicioide subclade. [file 1471-2229-10-183-S1.PDF]

Additional file 1

Table S1. GenBank accession numbers for three chloroplast gene regions used to estimate the age of separation of different species of the vicioide subclade.

Gene	*Medicago truncatula*	*Melilotus alba*	*Trifolium*	*Pisum sativum*	*Vicia*	*Albizia*
rbcL	AC093544	DQ006095	*T. pratense* AY395564	X03853	*V. cracca* AY395566	*A. julibrissin* Z70147
matK	AF522109	AF142738	*T. albopurpureum* AF522116	EU307313	*V. hirsuta* AF522157	*A. versicolor* AF274210
trnL	AC093544	DQ311713	*T. praetermissum* DQ311887	EF010972	*V. nipponica* AY839407	*A. julibrissin* EU439984
