# Supplementary material for: Medicago truncatula contains a second gene encoding a plastid located glutamine synthetase exclusively expressed in developing seeds
Source: BMC Plant Biol. 2010 Aug 19;10:183. doi: 10.1186/1471-2229-10-183 (PMC3095313; doi:10.1186/1471-2229-10-183)
Supplement: Additional file 2 — List of primers. Sequence of the primers used to amplify GS2 genes from Medicago truncatula and Melilotus alba. [file 1471-2229-10-183-S2.PDF]

Additional file 2

Table S1. Primers used to amplify *GS2b* genes from *M. alba*

	Forward (5'- 3')		Reverse (5'- 3')
Gs2F1	GCAGATAAGTCATTTGGA	Gs2R1	ATGTGGAyCTTGTGGCGA
Gs2F2	GGGCTTCAAGGTACATCC	Gs2R2	TCAAATCCTCCATCTTCC

Table S2. Primers used for the 5'RACE, 3' RACE and full length *MtGS2b* transcript amplification.

		Forward (5'- 3')	Reverse (5'- 3')
**5'RACE**	outer	Ambion's 5′ RACE outer primer	CTTATTTGTAGGGATAGGCTCACC
	inner	Ambion's 5′ RACE inner primer	GCTTGTCCAGTGCTAGATCCATC
**3'RACE**	outer	TGAGGGAAGATGGAGGATTTGAG	Ambion's 3′ RACE outer primer
	inner	GAATCTATCCCTTCGCCACAAG	Ambion's 3′ RACE inner primer
**Full-length**		ATAATCACTTTCATAGCCTCTAAAGG	CAATGTAACTCTGTCCATACC

Table S3. Primers used for RT-PCR

	Forward (5'- 3')	Reverse (5'- 3')
MtGS2b-α	GTGGTTACGTTTATTTAATACCATTGTC	GCCAGAGTTGATTGCCATTGC
MtGS2b-β	ATCTGGTGTCTGACACAGCAAAAC	GCCAGAGTTGATTGCCATTGC
MtGS2a	TCACTTGAACCCATTTCCTAAG	CCAGAGTTGACTGCCATTAC
Elf1-α	CCACCAACCTTGACTGGTAC	CCACGCTTGAGATCCTTCAC
